# Activation of *PTGS2*/NF‐κB signaling pathway enhances radiation resistance of glioma

**DOI:** 10.1002/cam4.1971

**Published:** 2019-02-10

**Authors:** Cheng Tan, Liang Liu, Xiaoyang Liu, Ling Qi, Weiyao Wang, Guifang Zhao, Libo Wang, Yimeng Dai

**Affiliations:** ^1^ Department of Neurology China‐Japan Union Hospital of Jilin University Changchun, Jilin China; ^2^ Department of Radiology China‐Japan Union Hospital of Jilin University Changchun, Jilin China; ^3^ Department of Pathophysiology Jilin Medical University Jilin China

**Keywords:** glioma, NF‐κB signaling pathway, *PTGS2*, radiation resistance

## Abstract

**Objective:**

We focused on the effects of *PTGS2*/NF‐κB signaling pathway on the radiation resistance of glioma in the study.

**Methods:**

We downloaded the microarray data from the Gene Expression Omnibus (GEO) database. We verified transfection successfully through QRT‐PCR analysis. Immunofluorescence was used to detect γH2AX content under 2 Gy radiation. The survival rates of cells under 2 Gy irradiation were tested by clonogenic survival assay. Flow cytometry was used to detect cell cycle. Western blot was applied to detect the expression of NF‐κB pathway‐related proteins. We also used MTT assay to detect the proliferation of cells.

**Results:**

In this research, we discovered that the expression of the *PTGS2* was upregulated in radiation‐resistant glioma cells. The radio‐tolerance rate of U87 cells was obviously elevated after the overexpression of *PTGS2*. The radioresistance of U87R cells was significantly reduced after the knockdown of *PTGS2*. After radiotherapy, the number of cells arrested in G2/M phase decreased after *PTGS2 *overexpression in U87cells but increased in PTGS2 knockdown in U87R cells. The survival rate of U87 and U87R cells under radiation decreased significantly after the addition of NF‐κB inhibitor. The proliferation of U87 cells was suppressed by radiation and the addition of Bay 11. In addition, *PTGS2* activated NF‐κB signaling pathway and prevented DNA damage after radiotherapy. Lastly, *PTGS2* was proved to facilitate tumor cell proliferation and improve the radio‐tolerance.

**Conclusion:**

*PTGS2*/NF‐κB signaling pathway was involved in radio‐tolerance of glioma cells, which provided a new insight into glioma therapy.

## 1 INTRODUCTION

Glioma is the most common malignant cancer in the central nervous system in adults. Most patients with glioma could live for only a few months without treatment.^1^ Patients with glioma have a median survival time that rarely exceeds 18 months in both children and adults. It is critical to identify novel treatment options for patients with glioma.^2^ There are many methods for the treatment of glioma such as surgery, radiotherapy, chemotherapy, and a combination of different modalities. Radiotherapy has already become one of the most important ways in tumor therapeutic strategy.

Radiotherapy is a common treatment method for patients with various types of cancer. Reactive oxygen species produced in radiotherapy induces a variety of DNA lesions, including oxidized base damage, single‐strand breaks (SSBs), and double‐strand breaks (DSBs). These lesions may result in cell death through mitotic catastrophe and apoptosis.^3^ Radiation may upregulate or downregulate the expression of several repair genes. Changes in the expression of these genes can affect the ability of cells to repair DNA damage.^4^ Meanwhile, radiation tolerance is still a problem for radiotherapy. Radiation can cause DNA damage and induce endoplasmic reticulum stress (ERS), while sustained ERS protects cells from death and induces treatment resistance via regulation of the expression of apoptosis‐ and cell cycle‐related proteins.^5^ The ERS signaling pathway protein kinase RNA‐like endoplasmic reticulum kinase (PERK) regulated radioresistance in carcinoma through nuclear factor‐κB (NF‐κB)‐mediated phosphorylation of eukaryotic initiation factor‐2 (*eIF2α*), enhancing X‐ray‐induced activation of DNA DSB repair, cell apoptosis inhibition, and G2/M cell cycle arrest.^6^

NF‐κB signaling is critically important during glioma development and progression. Using specific inhibitors to inhibit the activation of NF‐κB could inhibit glioma growth.^1^ It was reported that NF‐κB activation in response to chemotherapeutic agents protected U87 glioma cells in vitro.^7^ The transcription factor NF‐κB plays a critical role in inflammation, oncogenesis, and tumor progression. Its family includes p65 (RelA), RelB, c‐Rel, p50/p105 (NF‐κB1), and p52/p100 (NF‐κB2).^8^, ^9^ NF‐κB is a nuclear transcription regulatory factor with specific DNA binding sequences participating in cellular responses to ionizing radiation.^10^ NF‐κB regulates the radioresistance by regulating radiation‐induced DNA DSBs repair and cell cycle arrest.^11^, ^12^, ^13^ TNF‐α induces expression of NF‐κB target genes, such as genes related to proliferation (PGs2, Cyclin D1, and c‐Myc), anti‐apoptosis (Bcl‐2, cIAP‐1, and survivin), invasion (MMP‐9 and ICAM‐1), and angiogenesis (VEGF). The NF‐κB signaling pathway plays a pivotal role in regulating the immune response, inflammation, and oncogenesis.^14^ However, the mechanism of NF‐κB on radiotherapy still remains unclear.

Prostaglandins (PGs) play an important role as mediators in physiology. PGs have also been implicated in cancer development.^15^ The rate‐limiting enzyme in the synthesis of PGs from arachidonic acid is PTGS1 and PTGS2. Recent studies have shown *PTGS2* expression to be upregulated in a number of human tumor types.^16^, ^17^ Inhibitors of *PTGS2* have been reported to reduce the proliferation rate of tumor cell lines grown and acted as radiosensitizing agents.^18^ NF‐κB initiates the inflammatory response by regulating the expression of PGS2 and other cytokines.^19^ As a result, we suppose that NF‐κB affecting radiation resistance in tumor is via regulation of *PTGS2*.

In this study, we linked *PTGS2* with NF‐κB signaling pathway, which is closely related to cancer cell proliferation and radiotherapy tolerance. Here, we sought to investigate *PTGS2* upregulation to explore the ability of NF‐κB signaling on the regulation of glioma cell activities. We hypothesized that NF‐κB signaling pathway could influence radiotherapy tolerance of glioma cells through regulating *PTGS2*, which may imply potential therapeutic approaches for the treatment of glioma.

## 2 METHODS

### 2.1 Bioinformatic analysis

Gene expression data (Accession No. GSE82139) were retrieved from GEO (https://www.ncbi.nlm.nih.gov/geo/). This chip contains the following subjects such as PG35s and PG35 which were GICs and non‐GICs cultures derived from a glioblastoma patient, respectively, and irradiated group was irradiated with fractionated. We used R software to screen out differentially expressed genes (DEGs) between radiation‐resistant samples and control samples. The Search Tool for the Retrieval of Interacting Genes database (STRING) was used to predict protein‐protein interactions of glioma radioresistance‐related genes obtained from PolySearch2. We used GSEA v3.0 software Becton Dickinson (Cambridge, MA, USA) to upload and analyze the enrichment of the differentially expressed genes. Default weighted enrichment statistic to process data for 1000 times with normalized *P* < 0.05 was adopted. Then, we used “ggplot2” package to operate graphic processing of the selected seven highest upregulated and downregulated results of GSEA reports. Then, we employed Cytoscape software to establish pathways network. “joyplot” and “dotplot” function was also adopted to visualize the distribution.

### 2.2 Cell model establishment and cell culture

Glioma cell line U87 was purchased from BeNa Culture Collection (Beijing, China) and cultured in 90% high sugar DMEM medium with 10% FBS (Invitrogen, Carlsbad, CA, USA) in a humidity atmosphere with 5% CO_2_ at the temperature of 37°C. For cell transfer, the cells were rinsed with Ca^2+^‐ and Mg^2+^‐free phosphate‐buffered saline (PBS; Sigma‐Aldrich, St. Louis, MO, USA) and dispersed with 0.25% trypsin solution containing 0.5 mmol/L ethylenediaminetetraacetic acid (Sigma‐Aldrich). The U87 cells were exposed to irradiation (IR, 2 Gy/d) for 7 days, and the surviving cells were cultured to establish radiation‐resistant cell line U87R. To establish radioresistant U87 cell line,^20^ cells were irradiated with X‐rays at a dose rate of 1.55 Gy/min. The doses used were as follows: 2, 4, 6, and 8 Gy for clonogenic survival assays and 2 Gy for MTT assays, cell cycle analysis, immunofluorescence, Western blot analysis. The dose of 2 Gy was selected from the results of a clonogenic survival assay.

### 2.3 QRT‐PCR assay

We extracted total RNA from cells using Trizol method, and cDNA was synthesized from extracted RNA using first‐strand synthesis kit (Thermo Fisher, Waltham, MA, USA) before QRT‐PCR. Assays were performed according to SYBR Green PCR Master Mix (Thermo Fisher) protocol. Gene transcript levels of each group were calculated using the 2^−∆∆CT^ method. β‐actin acted as the internal reference for PCR amplification, and predesigned primers used were listed in Table 1.

**Table 1:** The primer sequences for QRT‐PCR

	Primer sequences (5'‐3')
PTGS2 Forward	TAGGATTCAGGGCTTTCACTGGCT
PTGS2 Reverse	TGTCAGCCGACAATGAGATGTGGA
β‐actin Forward	GCACCACACCTTCTACAATG
β‐actin Reverse	TGCTTGCTGATCCACATCTG

### 2.4 Cell transfection

The plasmids pcDNA 3.1‐PTGS2, siPTGS2 were all provided by GenePharma (Shanghai, China). Before transfection, 1 × 10^5^ glioma cells (U87 and U87R cell lines) were cultured in 24‐well plates with complete medium for 24 hours. We used Lipofectamine 2000 (Invitrogen) to transfect the constructs into glioma cells that were cultured in serum‐free DMEM medium according to manufacturer's protocols. Stably expressed strains were obtained by culturing the transfected cells for 36 hours with 1 μg/mL puromycin.

### 2.5 Clonogenic survival assay

A standard colony formation assay was performed immediately after exposing the cell lines to X‐rays. After irradiation, the cells were trypsinized, suspended in PBS on ice, and plated on a 60‐mm dish (Falcon; Becton Dickinson, Franklin lake, NJ, USA). Colonies were fixed and stained 2 weeks later. Three replicates were used for each dose. Colonies with more than 50 cells were scored as survivors. Doses used were 0, 2, 4, 6, and 8 Gy. Three independent experiments were performed for each dose.

### 2.6 Cell cycle analysis

Evaluation of cell cycle phase distribution was performed using BD FACSCalibur flow cytometer (BD BioSciences, San Jose, CA, USA) and BD CellQuest Pro software (version 5.1; BD BioSciences). The treatment protocols were essentially the same as in the clonogenic survival experiments. All of the cultures were subconfluent at the time of collection. Cultures were collected for fixation with ice‐cold 70% ethanol, stained with propidium iodide, and analyzed using the same flow cytometer. Attached cells (those remained adhered to the dish) and floating cells (those detached from the monolayer) were stained separately.

### 2.7 Western blot

Cells were washed twice with ice‐cold PBS, and total protein was obtained using RIPA lysate through centrifugation at 18000 *g*, 4°C for 25 minutes. Proteins (70 mg for each group) were separated by 12% SDS‐PAGE, after electrophoresis, they were electroblotted onto PVDF membranes and blocked at room temperature using 5% non‐fat milk. Membranes were washed in tris buffer saline–Tween 20 (TBST) and incubated with primary antibodies for 40 minutes: anti‐ PTGS2 (rabbit polyclonal to PTGS2 antibody; 1:500; ab15191; Abcam, Cambridge, MA, USA), anti‐Bcl‐2 (rabbit monoclonal to Bcl‐2 antibody; 1:500; ab32124; Abcam), anti‐p50 (rabbit monoclonal to p50 antibody; 1:500; ab32360; Abcam), and anti‐CCNDBP1(rabbit polyclonal to CCND1 antibody; 1:500; ab220275; Abcam). Membranes were washed in TBS‐T three times (15 min/time) and incubated for 1 hour with horseradish peroxidase (HRP)‐labeled goat anti‐rabbit IgG secondary antibody (1:5000; ab205718; Abcam). Then, membranes were then washed twice with TBST for 10 minutes and exposed to chemiluminescence (ECL; Thermo Fisher). Visualization and quantification were performed using a microscope (Bio‐Rad, Hercules, CA, USA). β‐actin was used as the control to compare with proteins.

### 2.8 Immunofluorescence of γH2AX

To assess DNA damage, cells grown on glass coverslips were irradiated with 2 Gy for 24 hours. At this time point, cells were fixed in freshly prepared 4% paraformaldehyde solution for 10 minutes. at room temperature. For staining, the fixed cells were treated with 0.1% Triton X‐100 and incubated with anti‐γH2AX antibody (1:500; Abcam) at room temperature followed by the incubation with secondary Cy‐3 conjugated antibody (Jackson Immuno Research Laboratories Inc, West Grove, PA, USA). Slides were mounted in prolonged antifade solution supplemented with DAPI (Sigma‐Aldrich). We numbered the cells with over 50 foci after cells were irradiated to 2 Gy for 24 hours. All procedures were operated in triplicate. Images were collected using a laser scanning confocal microscope (MRC‐1024; Bio‐Rad).

### 2.9 MTT assay

After cells were irradiated and cultured for 0, 24, 48, and 72 hours, respectively, 20 μl tetrazolium salt 3‐(4,5‐dimethylthiazol‐2‐yl)‐2,5‐diphenyltetrazolium bromide (MTT; Sigma‐Aldrich) was seeded in each experimental group. After 4‐hours incubation at 37°C, in 5% CO_2_, the medium was substituted with 100 μL DMSO. Finally, the absorbance values of cell lysates at 570 nm were measured using a microplate reader (Thermo Fisher).

### 2.10 Luciferase reporter assay

We used Lipofectamine 2000 (Invitrogen) to transfect the luciferase reporter plasmids or empty vectors into cells together with the encoding gene plasmids. When transfecting glioma cells (both U87 and U87R cell lines) with luciferase reporter plasmids, we also set up a control group in which the cells were transfected with β‐galactosidase (β‐gal) expression vectors (Ambion, Austin, TX, USA). Luciferase activities were measured at 48 hours after transfection using a luciferase assay kit (Promega, Madison, WI, USA) following the protocol.

### 2.11 Statistical analysis

GraphPad 6.0 software (GraphPad Software, La Jolla, CA, USA) was used for statistical analysis. For comparisons between two samples, unpaired two‐tailed *t* tests were performed. Differences with a *P* value smaller than 0.05 were considered statistically significant. The data were documented as means ± SD.

## 3 RESULTS

### 3.1 *PTGS2* and NF‐κB signaling pathway were involved in the radioresistance of glioma

**Figure 1:**
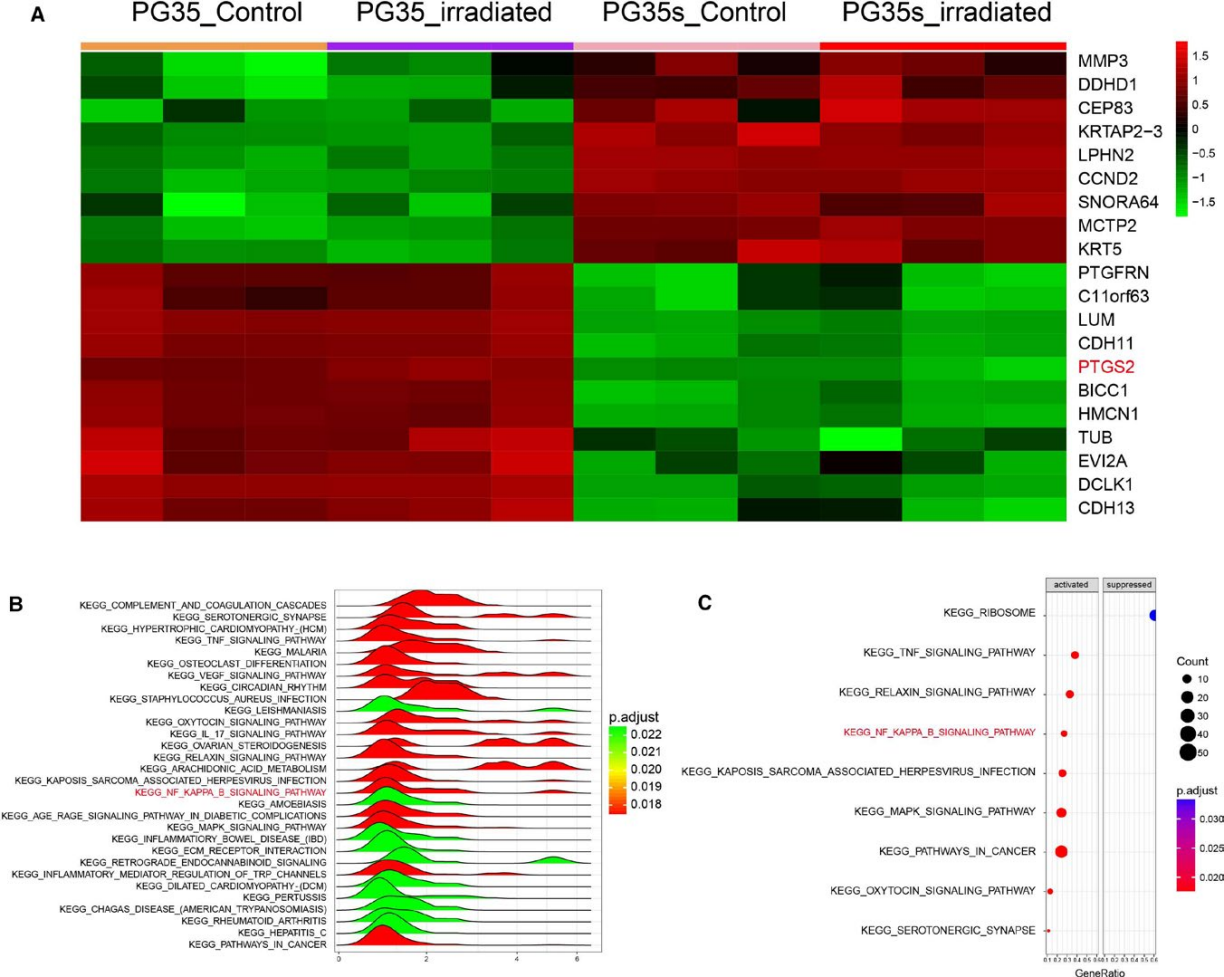
Bioinformatics analysis of glioma radiotherapy tolerance. A, Hierarchical cluster analysis of the upregulated and downregulated mRNAs. In the heat map, green color represents downregulation whereas red represents upregulation. B and C, Joyplot and dotplot results of the dysregulated KEGG pathways in glioma. In the ridge plot (B), the color was applied according to the adjusted p value. Every ridge represents a pathway. When a ridge was on the right side of 0, the pathway was activated in glioma. In the dotplot (C), activated and suppressed columns mean activated and suppressed in glioma. *P*.adjust: adjusted *P* value

**Figure 2:**
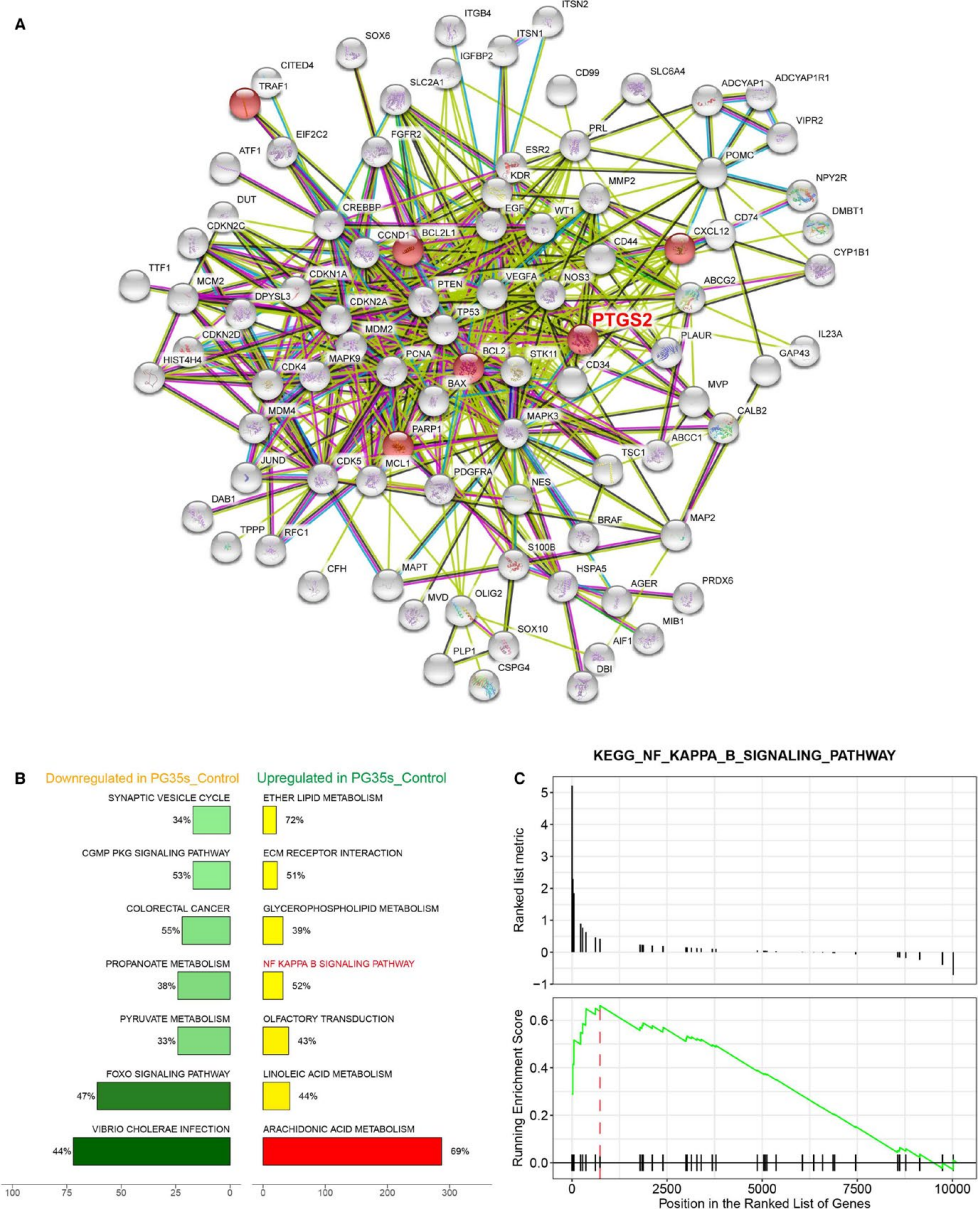
Bioinformatics analysis of NF‐κB signaling pathway. A, Protein‐protein interaction networks of differentially expressed genes in glioma. This network was obtained from STRING analysis. PTGS2 was seen interacted with a plenty of proteins. B, A plot of seven most enriched KEGG pathways in PG35s. Pathways were ordered by normalized enrichment score (NES). Percentage beside the bar indicated the proportion of differential genes in pathway gene set. The x‐axis stands for the number of genes in a pathway. C, Gseaplot showed that most genes of NF‐κB signaling pathway were overexpressed in PG35s

Heat maps are typically used in molecular biology to represent the level of expression of many genes across a number of comparable samples. Top 10 upregulated and downregulated genes were shown in the heat map, and *PTGS2* was found among the upregulated genes in radioresistant groups (Figure 1A). The STRING analysis results showed that PTGS2 was involved in a plenty of PPI networks (Figure 2A), suggesting its potential involvement in the radioresistance of glioma. We then interrogated these differentially expressed genes to KEGG pathway analysis, and the results demonstrated that NF‐κB signaling pathway was significantly activated in radioresistant groups (Figures 1B,C and 2B,C). In conclusion, *PTGS2* might be involved in the radioresistance of gliomas.

### 3.2 The radio‐tolerant U87R cell model was successfully established

**Figure 3:**
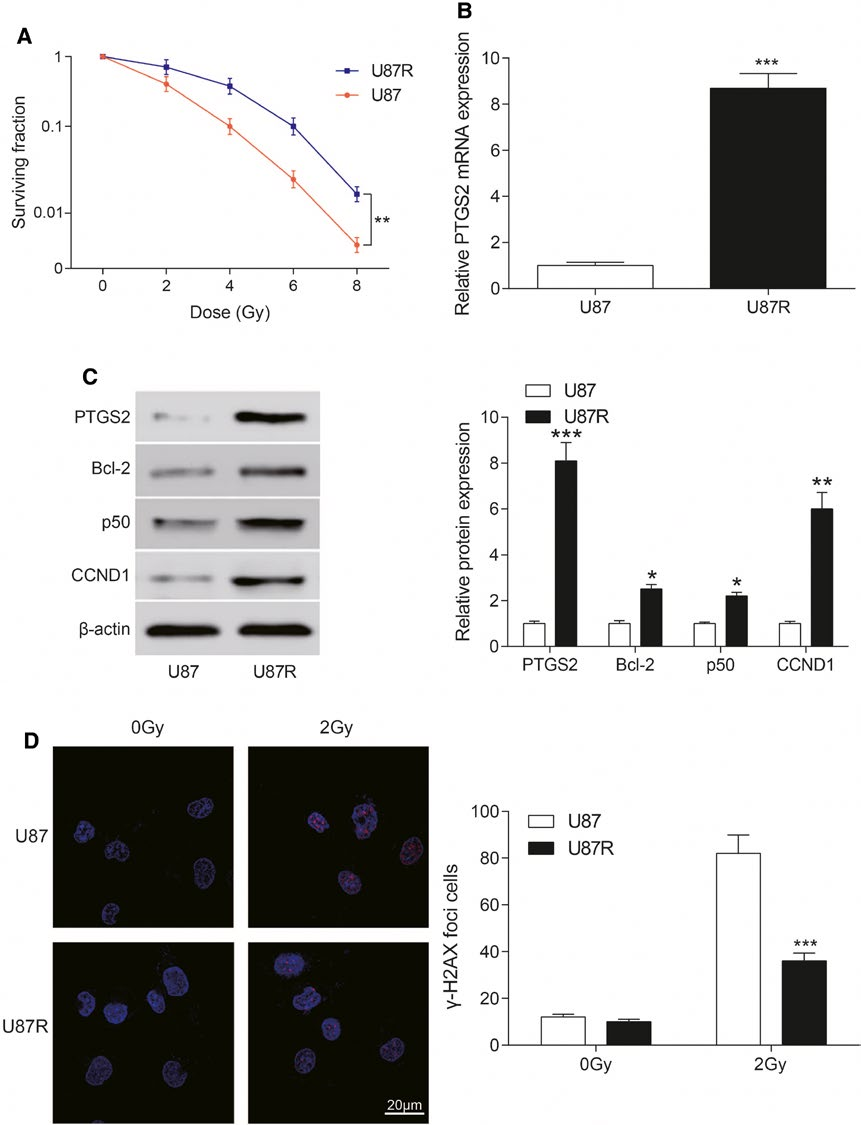
U87R cell radiotherapy tolerance. A, Survival fraction of U87R cells and U87 cells under different doses of irradiation was showed. **P < 0.01, compared with U87 cells. B, The contents of *PTGS2* mRNA in U87 and U87R cells were detected by QRT‐PCR. The expression of *PTGS2* mRNA in U87R cells was higher than that in U87 cells. ***P < 0.001, compared with U87 cells. C, The expression of related proteins (including PTGS2, Bcl‐2, p50 and CCND1) in NF‐κB signaling pathway was detected by Western blot. *P < 0.05, **P < 0.01, ***P < 0.001, compared with U87 cells. D, γ‐H2AX content was detected by immunofluorescence. γ‐H2AX content was significantly less in U87R cells under 2 Gy irradiation. ***P < 0.001, compared with U87 cells. Scale bar: 20 μm. Red dots were γ‐H2AX

After 2 Gy/d irradiation for 7 days, the surviving U87 cells were cultured continually to obtain radiation‐resistant cell lines. U87R cells showed higher survival rate compared with U87 cells after radiation through colony survival assay after same intensity of radiation (*P* < 0.01, Figure 3A). The expression of *PTGS2* mRNA in U87R cells was higher than that in U87 cells detected by PCR assay (*P* < 0.001, Figure 3B). The expressions of related proteins in NF‐κB signaling pathway were detected by Western blot. Compared with U87 cells, the expression of PTGS2, Bcl‐2, p50, and CCND1 in U87R cell line was significantly higher than that in U87 cell line (all *P* < 0.05, Figure 3C). γ‐H2AX (red fluorescence) was detected by immunofluorescence. The number of cells with greater than 50 foci of γ‐H2AX was significantly bigger in U87 cells compared with U87R cells (*P* < 0.001, Figure 3D), indicating that DNA of U87 cells was damaged more significantly than that of U87R cells after same intensity of radiation. All the results indicated that radiotherapy tolerant cell model U87R was successfully established.

### 3.3 Effects of PTGS2 on radiotherapy

**Figure 4:**
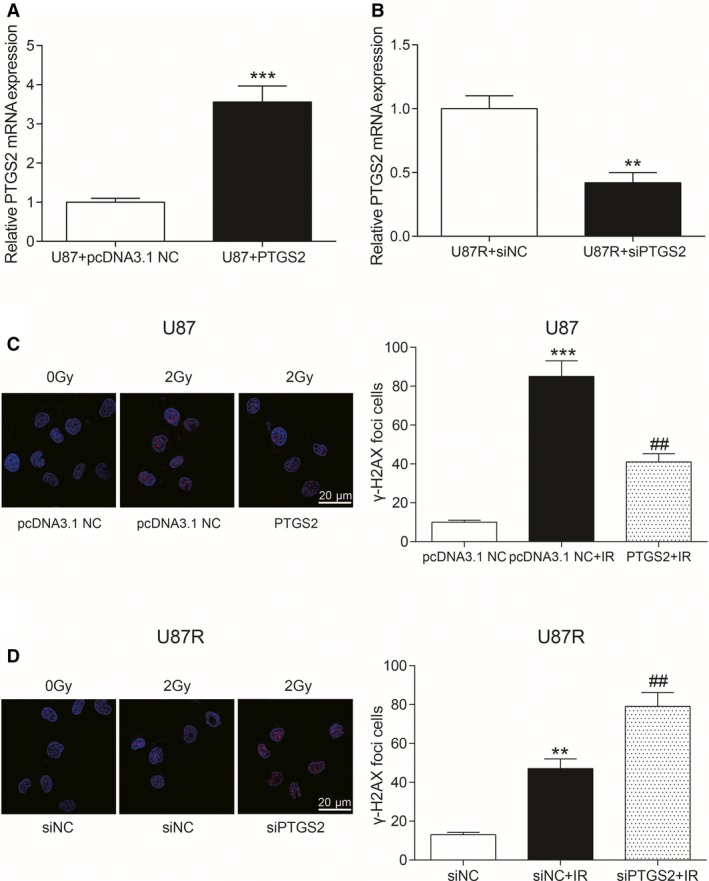
Effects of *PTGS2* on radiotherapy. A, The expression of *PTGS2* mRNA in U87 cells transfected with *PTGS2* overexpression plasmids and control group. ***P < 0.001, compared with U87 + pcDNA3.1 NC group. B, The expression of *PTGS2* mRNA in U87R cells transfected with siPTGS2 and the control group. **P < 0.01, compared with U87R + siNC group. C, Immunofluorescence was used to determine the content of γ‐H2AX in U87 cells. Scale bar: 20 μm. Red dots were γ‐H2AX. ***P < 0.001, compared with pcDNA3.1 NC group. ##*P* < 0.01, compared with pcDNA3. 1 NC + IR group. D, Immunofluorescence was used to determine the content of γ‐H2AX in U87R cells. Scale bar: 20 μm. Red dots were γ‐H2AX. **P < 0.01, compared with siNC group. ##*P* < 0.01, compared with siNC + IR group

The expression of *PTGS2* mRNA in U87 cells that were transfected with *PTGS2* overexpression plasmids increased significantly compared with U87 + pcDNA3.1 NC (*P* < 0.001, Figure 4A). The expression of *PTGS2* mRNA in U87R cells transfected with siPTGS2 decreased significantly compared to U87 + siNC (*P* < 0.01, Figure 4B). After *PTGS2* was overexpressed, the number of γ‐H2AX accumulation in cells was smaller than that of pcDNA3.1 NC group and pcDNA3.1 NC + IR group after the same intensity of radiotherapy (all *P* < 0.01, Figure 4C). After transfection with siPTGS2, γ‐H2AX accumulated in cells after radiotherapy was much more than that of siNC group and siNC + IR (all *P* < 0.01, Figure 4D). In conclusion, the expression of *PTGS2* mRNA played a positive role in preventing DNA damage in U87 cells after radiotherapy.

### 3.4 Effects of PTGS2 on radioresistance and cell cycle

**Figure 5:**
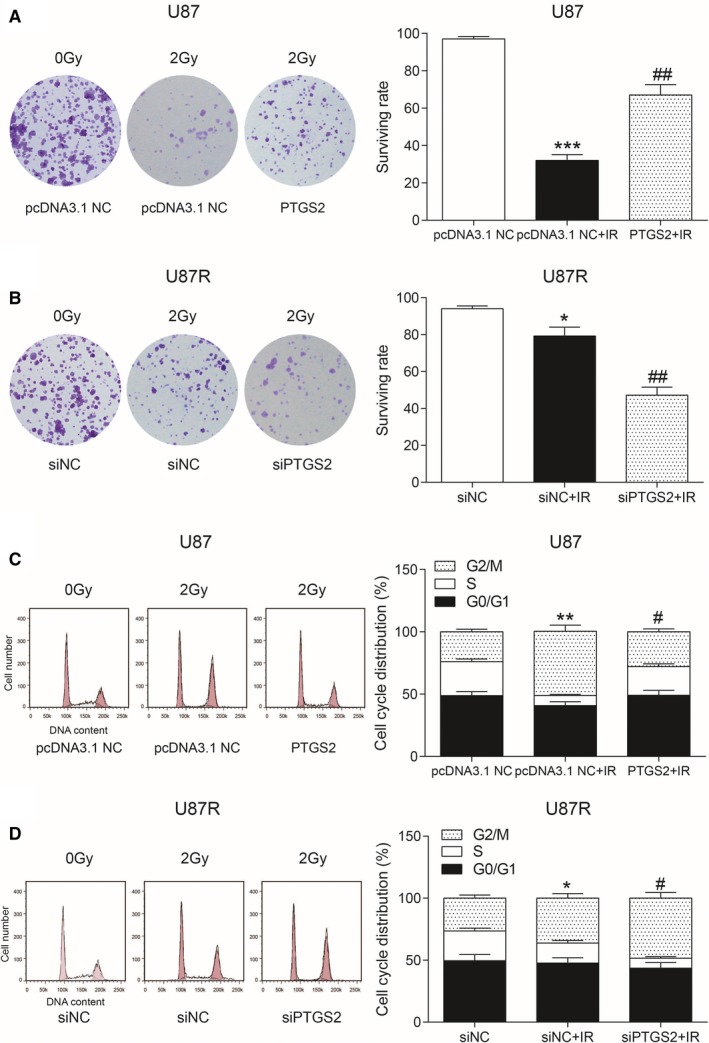
Effects of *PTGS2* on radioresistance and cell cycle. A, The survival rate of U87 cells was detected by clone survival assay. ***P < 0.001, compared with pcDNA3.1 NC group. ##*P* < 0.01, compared with pcDNA3. NC + IR group. B, The survival rate of U87R cells was detected by clone survival assay. *P < 0.05, compared with siNC group. ##*P* < 0.01, compared with siNC + IR group. C, Flow cytometry was used to detect U87 cell cycle. **P < 0.01, compared with pcDNA3.1 NC group. #*P* < 0.05, compared with pcDNA3.1 NC + IR group. D, Flow cytometry was used to detect U87R cell cycle. *P < 0.05, compared with siNC group. #*P* < 0.05, compared with siNC + IR group

The survival rate of U87 cells was detected by clone survival assay. The surviving rate of U87 cells was significantly higher after the overexpression of *PTGS2* compared to pcDNA3.1 NC group and pcDNA3.1 NC + IR group (all *P* < 0.01, Figure 5A). The survival rate of U87R cells was significantly reduced in radioresistance after knockdown of *PTGS2* compared with siNC group and siNC + IR group (all *P* < 0.05, Figure 5B). We used flow cytometry to detect U87 cell cycle. After radiotherapy, cells arrested in G2/M phase in PTGS2‐transfected group were less than that in pcDNA3.1 NC group and pcDNA3.1 NC + IR group (all *P* < 0.05, Figure 5C). The cells arrested in G2/M phase were significantly more in siPTGS2 group compared with siNC and siNC + IR group (all *P* < 0.05, Figure 5D). Therefore, *PTGS2* played an active role in radiotherapy tolerance.

### 3.5 Effects of NF‐κB signaling pathway on radiotherapy

**Figure 6:**
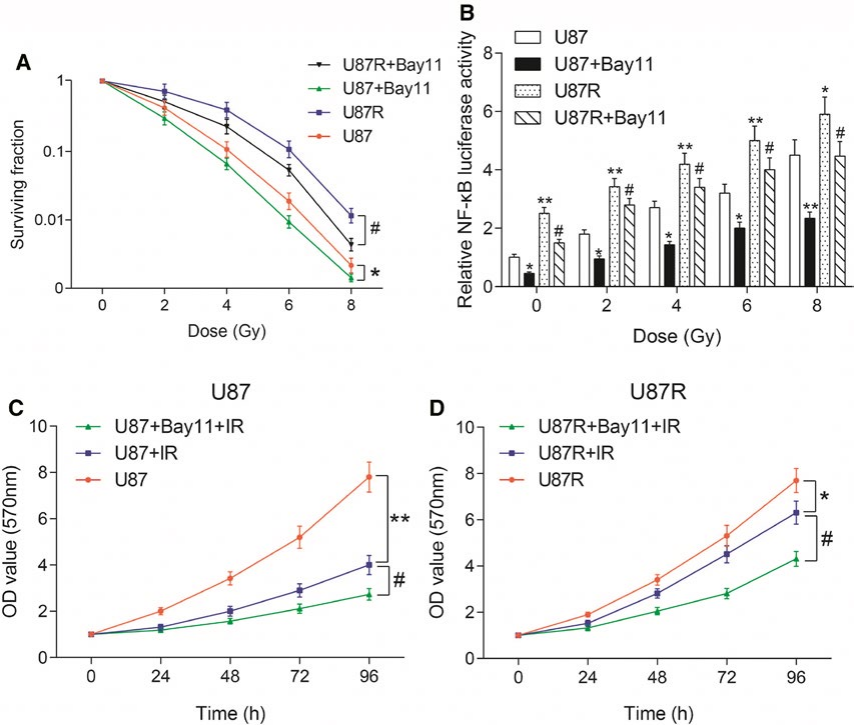
Effect of NF‐κB signaling pathway on radiotherapy. A, Survival rate was tested by clone survival assay. *P < 0.05, compared with U87 group. #*P* < 0.05, compared with U87R group. B, NF‐κB was detected by luciferase assay. *P < 0.05, **P < 0.01, compared with U87 group. #*P* < 0.05, compared with U87R group. C, The proliferation of U87 cells after radiotherapy was detected by MTT assay. **P < 0.01, compared with U87 group. #*P* < 0.05, compared with U87 + IR group. D, The proliferation of U87R cells after radiotherapy was detected by MTT assay. *P < 0.05, compared with U87R group. #*P* < 0.05, compared with U87R + IR group. Bay 11 was a NF‐κB signaling pathway inhibitor

Survival rate was tested by clone survival assay. The radiotherapy survival rate of U87 and U87R cells decreased significantly after the addition of NF‐κB inhibitor Bay 11 compared with U87 group and U87R group (all *P* < 0.05, Figure 6A). NF‐κB was detected by luciferase assay. After transfection with pNF‐κB‐luc plasmids, the expression of NF‐κB increased with the increase in radiotherapy. NF‐κB expression decreased after adding Bay 1, a NF‐κB inhibitor compared with U87 group and U87R group (all *P* < 0.05, Figure 6B). The proliferation of U87 cells after radiotherapy was detected by MTT assay. The proliferation of U87 cells after radiotherapy decreased, and adding Bay 11 lowered the proliferation compared with U87 group and U87 + IR group (all *P* < 0.05, Figure 6C). The proliferation of U87R cells after radiotherapy was detected by MTT assay. The results were similar to U87 cells (all *P* < 0.05, Figure 6D). We thus concluded that NF‐κB signaling pathway was activated in radiotherapy tolerance.

## 4 DISCUSSION

Radiation therapy is a common treatment method for cancer, whereas radioresistance of cancer cells is a huge clinical problem.^21^ Tumor radioresistance is a major reason for decreased efficiency of cancer radiation therapy.^22^ Through the bioinformatics analysis of differential gene expression, *PTGS2* was found highly expressed in radioresistant groups. We hypothesized that *PTGS2* was involved in the radioresistance of glioma, and subsequent experiments were carried out. We linked *PTGS2* with NF‐κB signaling pathway, which is closely related to cancer cell proliferation and radio‐tolerance and we find that *PTGS2* influenced radiotherapy tolerance of U87 cells through the NF‐κB signaling pathway.

In this study, we found that the radio‐tolerance of U87 cells was significantly increased after the overexpression of*PTGS2* compared with the group in which *PTGS2* was knocked down. *PTGS2* was reported to be overexpressed in cancer cells and associated with the resistance of cancer cells to IR.^23^ Eberhart et al find that *PTGS2* was upregulated in a number of human cancers.^16^, ^17^ Petersen et al^18^ found that inhibitors of *PTGS2* could act as radiosensitizing agents and reduce the proliferation rate of tumor cell lines. While Inoue et al claimed that *PTGS2* inhibitors enhanced the effects of radiotherapy on prostate cancer and largely prevented the stimulation of F98 cell infiltration into the brain,^24^, ^25^ Yang et al^26^ found that celecoxib, a *PTGS2*‐selective inhibitor, sensitized the responsiveness of these medulloblastoma cells to IR exposure in vitro. *PTGS2*‐induced radioresistance was negatively regulated through the phosphorylation of p38 at Tyr182 and that the phosphorylation of p38 induced by TNF‐alpha reduced the expression of Bcl‐2, BCL‐XL, but increased β‐catenin and E‐cadherin, leading to the decreased invasiveness of cells.^27^ Taken together, what we found was consistent with previous ones. *PTGS2* might play an active role in radiotherapy tolerance of cancer.

We investigated the effect of NF‐κB signaling pathway on radiotherapy by survival assay, luciferase assay, and MTT assay. We found that NF‐κB signaling pathway was activated in radiotherapy tolerance. NF‐κB was overexpressed in almost all cancer cells and mediated multiple signaling pathways to contribute to cell proliferation and to treatment resistance.^28^, ^29^, ^30^ INOS, PTGS2, and, TNF‐alpha, and, IL‐6 are important factors in the NF‐κB pathway.^31^ Ren et al found that radiation increased NF‐κB activity, while NF‐κB inhibitor enhanced radiation‐induced cell death. Blocking the NF‐κB pathway sensitized the radiosensitivity of HCC cells.^32^ NF‐κB in the irradiated cells determined radioresistance of the tumor cells.^33^ Wu et al^34^ found that it was via NF‐κB/HIF‐1 signaling pathway that cancer cell proliferation, migration, and radioresistance were regulated. Inhibiting the activation of NF‐κB might be an effective alternative therapy for inhibiting glioma growth.^1^ Those findings were consistent with what we found in our study. Collectively, NF‐κB signaling pathway is activated in radiotherapy tolerance. Using specific inhibitors could inhibit the activation of NF‐κB, therefore inhibiting glioma growth.

In our study, we proved that NF‐κB signaling pathway could lead to radiotherapy tolerance by modulating the expression of *PTGS2*. At the same time, we still had to consider our limitations. Firstly, U87 cells were the only cells we used in experiments. The effect of *PTGS2* in other glioma cell lines and animal models could be further explored. Secondly, we explored the effects of *PTGS2* on radiotherapy tolerance by NF‐κB signaling pathway through a limited number of experiments; thus, more experiments could also be conducted to explore the specific molecular mechanism.

In conclusion, our findings have demonstrated that *PTGS2* influenced radiotherapy tolerance of U87 cells through the NF‐κB signaling pathway. Importantly, we demonstrated that this pathway promoted the resistance to IR, which was a finding with important therapeutic implications for human glioma.

## CONFLICT OF INTEREST

The authors confirm that there are no conflicts of interest.
